# Classification of COVID19 Patients Using Robust Logistic Regression

**DOI:** 10.1007/s42519-022-00295-3

**Published:** 2022-09-21

**Authors:** Abhik Ghosh, María Jaenada, Leandro Pardo

**Affiliations:** 1 Indian Statistical Institute, Kolkata, India; 2 Department of Statistics and O.R., Complutense University of Madrid, Madrid, Spain

**Keywords:** Density power divergence, High-dimensional data, Sparse logistic regression, COVID-19, Gene expression

## Abstract

Coronavirus disease 2019 (COVID19) has triggered a global pandemic affecting millions of people. Severe acute respiratory syndrome coronavirus 2 (SARS-CoV-2) causing the COVID-19 disease is hypothesized to gain entry into humans via the airway epithelium, where it initiates a host response. The expression levels of genes at the upper airway that interact with the SARS-CoV-2 could be a telltale sign of virus infection. However, gene expression data have been flagged as suspicious of containing different contamination errors via techniques for extracting such information, and clinical diagnosis may contain labelling errors due to the specificity and sensitivity of diagnostic tests. We propose to fit the regularized logistic regression model as a classifier for COVID-19 diagnosis, which simultaneously identifies genes related to the disease and predicts the COVID-19 cases based on the expression values of the selected genes. We apply a robust estimating methods based on the density power divergence to obtain stable results ignoring the effects of contamination or labelling errors in the data and compare its performance with respect to the classical maximum likelihood estimator with different penalties, including the LASSO and the general adaptive LASSO penalties.

## Introduction

Coronaviruses (CoVs) are a group of enveloped, single, positive-stranded RNA viruses causing mild to severe respiratory illnesses in humans. Coronavirus disease 2019 (COVID-19), caused by the severe acute respiratory syndrome coronavirus 2 (SARS-CoV2), has lead to a global pandemic affecting millions of people and causing high mortality rates worldwide. Nonetheless, the actual knowledge about COVID19 is limited, and numerous studies have been carried out to identify genes involved in the host response to the SARS-CoV-2 infection, so as to determine mechanisms of pathogenicity and potential therapeutic targets (see, e.g., [20, 22, 24, 29] among many others). Viral infections of human cells lead to the production of interferons (IFNs) as an antiviral mechanism. In majority of cases, patients are asymptomatic or exhibit mild symptoms, whereas in more severe cases, patients may develop severe lung injury and death from respiratory failure. Moreover, SARS-CoV-2 is able to achieve high viral load even in the absence of symptoms, increasing its contagiousness.

Upper airway gene expression analysis can be performed for identification of transcriptional regulatory mechanisms involved in the host response to infection by SARS-CoV-2 and consequently help to distinguish between patients suffering from COVID19 and other viral or non-viral acute respiratory illness (ARIs). Genetic variation may contribute to disease largely through misregulation of gene expression. Metagenomic Next-Generation Sequencing (mNGS) is an useful tool providing clinically actionable information for predicting causes of an infection, evaluating infectious disease risk and successful diagnosing. Therefore, genetic information may be used to build novel respiratory diagnostics that integrate host transcriptional signatures of infection. Conversely, gene expression profiling involves a large number of features, often much larger than the sample size. High feature dimensionality and paucity of samples possess a challenge for predictive classification and marker identification methodologies. Therefore, techniques for high dimensional data analysis need to be applied.

Despite the potential of mNGS, it presents some crucial barriers, including data cleanliness. Contamination of samples during specimen collection is a large concern given the increased analytical sensitivity of mNGS in comparison with standard culture methods. Accordingly, robust statistical analysis appears appropriate for the classification of COVID19 patients and identification of genes involved in patient’s response to the infection using mNGS data.

Among the existing high dimensional statistical techniques, the regularized logistic regression model provides simultaneous gene identification and patient classification through a likelihood of suffering from the disease. The low dimensional logistic regression model has been widely used as a powerful classifier, but classical estimation design is ill-posed in the high dimensional set-up and regularized methods need to be applied. Regularization techniques assume that only a few number of explanatory variables are actually involved in the true model underlying the data, so they perform variable selection and parameter estimation by combining a model-based loss function with a penalization on the absolute value of the model parameters. Several penalties have been explored in the literature. The LASSO (Least Absolute Shrinkage and Selection Operator) procedure [27] stands within regularization methods, as it performs remarkably well as both a selector of important variables and as a prediction engine with computational feasibility. Later, Shevade and Keerthi [25] proposed sparse logistic regression based on the LASSO penalty and Cawley and Talbot [9] investigated sparse logistic regression with Bayesian penalty. However, it has been criticized for its biasedness, as it tends to select many noisy features with high probability, and consequently Zou [31] proposed the Adaptive-LASSO (referred as Ad-LASSO in the following) as an alternative to overcome this weakness. Huang et al. [17] applied adaptive LASSO to the logistic regression model and showed convenient asymptotic properties of the resulting estimators. Wide literature applies the regularized logistic regression model for gene identification and diagnosis of a disease though gene expression profiling. Some examples are Wu et al. [28], Jacob et al. [18] and Ghosh and Chinnaiyan [12]. In contrast, both LASSO and Adaptive LASSO procedures are based on the logistic likelihood function and hence, inherits severe lack of robustness; so both methods are sensitive to contamination in the sample.

While classical estimating methods are based on the maximum likelihood estimator (MLE), recent literature has shown the advantage of using divergence-based methods in terms of robustness, with an unavoidable (but often not quite significant) loss of efficiency. Robust methods for logistic regression based on bounded deviances have been introduced in Bianco and Yohai [4]. Cantoni and Ronchetti [8] studied robust M-estimators for generalized linear models and later Avella-Medina and Ronchetti [3] extended the theory for general penalized M-estimators in shrinking neighborhoods. Recently, Bianco et al. [5] studied penalized weighted M-estimators for the logistic regression model with random penalties. Basu et al. [6] introduced the minimum density power divergence (DPD) estimators for general statistical models, which are indeed robust against outliers and leverage points, fisher-consistent and enjoy asymptotic properties derived under much simpler conditions compared to the general M-estimators. The DPD has the interpretation of being a natural generalization of the likelihood-based loss function, so that the MLE is included as a particular case of the DPD-based family. Ghosh and Basu [13] studied the minimum DPD estimator (MDPDE) for generalized linear regression models, including the logistic regression, in the low-dimensional set-up, and later Basu et al. [7] extended the methodology for the high-dimensional logistic regression model, yielding the penalized MDPDE. They considered several penalty functions, including the LASSO and the Ad-LASSO penalties, as well as more general weighted adaptive LASSO (AW-LASSO) penalties. This last work [7] is particularly motivated from the excellent performances, both in terms of estimation accuracy and variable selection optimality, of the penalized DPD-based procedures observed under the high-dimensional linear regression model [14, 15].

In this paper, we propose to develop a COVID19 patients classifier through their upper airway gene expression using the penalized logistic regression model, which simultaneously carries out important gene selection. We apply different estimation methods, namely the classical MLE and the MDPDEs penalized with the LASSO, Ad-LASSO and AW-LASSO penalties (specific to the SCAD penalty of Fan and Li [10]) as developed in [7]. While adaptive penalties enhance the variable selection property, robust procedures based on the DPD have been shown to perform competitively with non-robust ones in the absence of contamination, and to improve the estimation accuracy and robustness in a contaminated scenario. Further, AW-DPD-LASSO estimator based on nonconcave penalties acquires many of its advantageous properties with less computational burden.

The outline of the paper is as follows. Section 2 introduces the minimum DPD estimator family and the corresponding penalized estimators. Section 3 describes the real dataset containing the upper airway host transcriptional response of patients suspected of suffering from COVID19 disease. Section 4 studies two classification problems, diagnosis of COVID19 and differentiation between COVID19 and other viral ARIs and discusses the performance of the logistic regression model fitted with different robust and non-robust estimators in both situations. In Sect. 5, some final conclusions are drawn.

## Robust Regularized Logistic Regression

Let us consider dichotomous and independent response variables $Y_1,..Y_n$, each independently following a Bernoulli distribution, as

$$\begin{aligned} P(Y_i = 1) = \pi _i,\quad i=1,...,n, \end{aligned}$$

where $\pi _i \in [0,1].$ The logistic regression model assesses the Bernoulli probabilities, $\pi _i,$ that are related to a fixed or random *k*-dimensional vector of explanatory variables, ${\varvec{x}}_i,$ through a common regression parameter $\varvec{\beta }\in {\mathbb {R}}^k$, for each $i=1, \ldots , n$, satisfying

$$\begin{aligned} \text {logit}(\pi _i) = {\varvec{x}}_i^T\varvec{\beta } \end{aligned}$$

where the function $\text {logit}(p) = \log \left( \frac{p}{1-p}\right) .$ For simplicity, here, we have assumed that the intercept term is included within the covariate vector ${\varvec{x}}$ (in its first component). Applying the inverse logit function, the logistic model gives the partnership class probability. In the following, we denote $\pi _i = \pi \left( {\varvec{x}}_i^T\varvec{\beta }\right) ,$ the probability of success of the response $Y_i,$ emphasizing its dependence of the observation ${\varvec{x}}_i$ and the regression parameter vector $\varvec{\beta }$. Therefore, to fit the logistic regression model it suffices to estimate the common parameter $\varvec{\beta }$ from the observed data.

As discussed in Sect. 1, classical estimation methods based on the likelihood function for the logistic regression model yield the MLE, known to be asymptotically efficient (is a BAN estimator) but not robust. Given the observed data $(y_1,{\varvec{x}}_1),..,(y_n,{\varvec{x}}_n),$ the MLE, $\widehat{\varvec{\beta }},$ is defined by

1 $$\begin{aligned} \widehat{\varvec{\beta }} = {\text {arg}} {\text {max}}_{\varvec{\beta }\in {\mathbb {R}}^k} {\mathcal {L}}(\varvec{\beta }), \end{aligned}$$

being

2 $$\begin{aligned} {\mathcal {L}}(\varvec{\beta }) = \prod _{i=1}^{n} \pi \left( {\varvec{x}}_i^T\varvec{\beta }\right) ^{y_i} \left( 1-\pi \left( {\varvec{x}}_i^T\varvec{\beta }\right) \right) ^{1-y_i} \end{aligned}$$

the likelihood function. Equivalently, the MLE can be obtained by minimizing the negative log-likelihood, $-\log \left( {\mathcal {L}}(\varvec{\beta })\right)$. We will adopt this last formulation, where the estimator is computed as the minimum of a so-called loss function. Then, to achieve robustness in the estimation, an alternative loss function must be used. In this line, Ghosh and Basu [13] presented a robust family of estimators for the generalized linear models based on the DPD approach and proved robustness its properties. In particular, given the observed data $(y_1,{\varvec{x}}_1),..,(y_n,{\varvec{x}}_n),$ the DPD for the logistic regression model yields

3 $$\begin{aligned} d_{\alpha }\left( \varvec{\beta }\right) =&\frac{1}{n^{1+\alpha }}\sum \limits _{i=1}^{n}\left\{ \left( \pi ^{1+\alpha }({\varvec{x}}_{i}^{T}\varvec{\beta }) +\left( 1-\pi ({\varvec{x}}_{i}^{T}\varvec{\beta })\right) ^{1+\alpha }\right) \right. \nonumber \\&-\left( 1+\frac{1}{\alpha }\right) \left( y_{i}\pi ^{\alpha }({\varvec{x}}_{i}^{T}\varvec{\beta }) +(1-y_{i})\left( 1-\pi ({\varvec{x}}_{i}^{T}\varvec{\beta })\right) ^{\alpha }\right) \nonumber \\&\left. +\frac{1}{\alpha }\left( y_{i}^{\alpha +1}+(1-y_{i} )^{\alpha +1}\right) \right\} . \end{aligned}$$

where the tuning parameter $\alpha \ge 0$ controls the trade-off between efficiency and robustness. The minimum DPD estimator, $\widehat{\varvec{\beta }}_\alpha ,$ (MDPDE) is defined as the minimizer of the loss function given in (3),

4 $$\begin{aligned} \widehat{\varvec{\beta }}_\alpha = {\text {arg}} {\text {min}}_{\varvec{\beta }\in {\mathbb {R}}^k} d_{\alpha }\left( \varvec{\beta }\right) . \end{aligned}$$

Furthermore, the DPD loss function can be defined at $\alpha = 0$ taking continuous limits, and the resulting MDPDE coincides with the MLE. That is, the proposed family of MDPDPE can be considered as a generalization of the MLE. The MDPDEs, $\widehat{\varvec{\beta }}_\alpha ,$ demonstrably enjoy great asymptotic properties although they entail an unavoidable loss of efficiency. Conversely, the gain in robustness is in many cases cost-effective.

On the other hand, dealing with high dimensional data requires additional assumptions on the model parameters. In particular, we assume that the true regression vector is assumed to be sparse, that is, having few non-null elements. Explanatory variables with zero regression coefficient are not significant for the model. Thus, variable selection needs to be performed jointly with the parameter estimation. Regularization methods are characterized by combining a loss function (from the model) and a penalty function that induces zero estimation of many coefficients.

The classical penalized (regularized) LASSO estimator of $\varvec{\beta }$ couples the negative loglikelihood loss function and the popular LASSO penalty,

5 $$\begin{aligned} \widehat{\varvec{\beta }}_{\text {LASSO}} = {\text {arg}} {\text {min}}_{\varvec{\beta }\in {\mathbb {R}}^k} \left[ - \log {\mathcal {L}}(\varvec{\beta }) + \lambda \sum _{j=1}^k |\beta _j|\right] \end{aligned}$$

where $\lambda$ is a regularization parameter controlling the shrinkage of the regression vector $\varvec{\beta }$. For more details, see Hastie et al. [16]. The choice of $\lambda$ then determines the sparsity of the model; the greater is $\lambda$, the greater the weight of the penalty in the objective function is. Several criteria for the election of the regularization parameter have been proposed in the literature, including cross-validation or information criteria adapted to the high dimensional set-up. Fokianos [11], Park and Hastie [21], Plan and Vershynin [23], Zhu and Hastie [30] and Sun and Wang [26] are interesting papers based on the LASSO estimator for the logistic regression model.

Basu et al. [7] extended the LASSO procedure with DPD-based loss function for the logistic regression model, producing more robust estimators. The so-called LASSO penalized MDPDE (DPD-LASSO) is then given by

6 $$\begin{aligned} \widehat{\varvec{\beta }}_{\alpha , \text {LASSO}} = {\text {arg}} {\text {min}}_{\varvec{\beta }\in {\mathbb {R}}^k} Q_\alpha \left( \varvec{\beta }\right) = {\text {arg}} {\text {min}}_{\varvec{\beta }\in {\mathbb {R}}^k} \left[ d_\alpha (\varvec{\beta }) + \lambda \sum _{j=1}^k |\beta _j|\right] . \end{aligned}$$

One of the major drawbacks of the LASSO penalty is that the estimators obtained with such penalty are not consistent, i.e., they lack the oracle property (Fan and Li [10]). Since LASSO function equally penalizes all the coefficients, it over-penalizes coefficients of irrelevant variables leading to a biased estimator. To overcome the bias deficiency, Zou [31] proposed the adaptive LASSO procedure in which adaptive weights are applied to different coefficients. Then, the adaptive LASSO objective function is given by

$$\begin{aligned} Q_\alpha \left( \varvec{\beta }\right) = - \log {\mathcal {L}}(\varvec{\beta }) + \lambda \sum _{j=1}^k \frac{1}{|{\widetilde{\beta }}_j|}|\beta _j|\end{aligned}$$

where $\widetilde{\varvec{\beta }} = \left( {\widetilde{\beta }}_1,...,{\widetilde{\beta }}_k \right)$ is a consistent estimator of $\varvec{\beta }.$ The initial estimator $\widetilde{\varvec{\beta }}$ weights the penalty to which each element of the estimated vector is subjected. For zero initially estimated elements, we can simply define a sufficiently great penalty bound. Therefore, lower elements in $\widetilde{\varvec{\beta }}$ entail a greater penalty, inducing the sparsity in the adaptive LASSO estimator and conversely lower weights are assigned to large initially estimated coefficients. This adaptive penalty reduces the bias problem of the standard LASSO. Some interesting results in relation to the adaptive LASSO estimator in logistic regression models can be seen in Algamal and Lee [1], Araveeporn [2], Bianco et al. [5] and references therein.

The idea of weighting the LASSO penalization can be extended to a more general framework, yielding the adaptive weighted LASSO estimator with objective function

7 $$\begin{aligned} Q_\alpha \left( \varvec{\beta }\right) = - \log {\mathcal {L}}(\varvec{\beta }) + \lambda \sum _{j=1}^k w(|{\widetilde{\beta }}_j|)|\beta _j|. \end{aligned}$$

An interesting proposal for the weighted function is the first derivative of the nonconcave SCAD penalty given by

8 $$\begin{aligned} w(s) = {\text {I}}(s\le \lambda ) + \frac{(a\lambda -s)_{+}}{(a-1)\lambda }{\text {I}}(s>\lambda ). \end{aligned}$$

with $a >2,$ where ${\text {I}}$ and $(\cdot )_{+}$ denote the indicator and positive part functions, respectively. The resulting weighted adaptive penalty is a linear approximation of the SCAD, and hence, it is expected to work as a substitute for this nonconcave penalty, improving unbiasedness, continuity and sparsity properties of the LASSO estimator. The weighted adaptive penalized estimator with this weight function will be referred to as the AW-DPD-LASSO.

The adaptive and weighted adaptive LASSO procedure can be easily adapted to the DPD-based loss function leading to an objective function of the form

9 $$\begin{aligned} Q_\alpha \left( \varvec{\beta }\right) = d_{\alpha }\left( \varvec{\beta }\right) + \lambda \sum _{j=1}^k w(|{\widetilde{\beta }}_j|)|\beta _j|. \end{aligned}$$

The minimization of the objective (9) produces robust adaptively weighted DPD-LASSO estimators, which includes the DPD-LASSO estimator for $w(\cdot ) = 1.$ The resulting penalized MDPDEs are indeed robust for all positives values of $\alpha$ when the initial estimator $\widetilde{\varvec{\beta }}$ is also robust, as proved in Basu et al. [7], and non-robust at $\alpha =0$ corresponding to the MLE. Moreover, they are consistent and asymptotically normal in the high dimensional data set-up with non polynomial order, i.e., when $\log (k) = O(n^s)$ for some $s\in (0,1),$ under some regularity conditions. Conversely, the gain in robustness entails an efficiency loss. Basu et al. [7] empirically compared the performance of the MDPPE for different values of $\alpha$ with high ultra-dimensional data, concluding that MDPDEs stand competitive in the absence of contamination and improve the model selection and classification rate in a contaminated scenario. The optimal value of the tuning parameter $\alpha$ directly depends on the data, as larger values of $\alpha$ produce more robust estimators which are preferable for high data contamination rate. Moderately large values of $\alpha$, over $0.3-0.5,$ have been recommended in the literature for worthwhile trade-off between robustness and efficiency.

## Data Description and Pre-processing

We study the upper airway host transcriptional response in patients with COVID19 ($n=93$), other viral ($n=41$) and non-viral ($n=100$) ARIs so as to identify genes involved in the host response on host and build a classifiers capable of pairwise differentiate between classes.

The data were first considered in Mick et al. [20] who conducted an observational cohort study at the University of California, San Francisco (UCSF) and Zuckerberg San Francisco General Hospital. They evaluated leftover RNA extracted from clinical swab specimens processed at the UCSF Clinical Microbiology Laboratory and performed a clinician-ordered test for SARS-CoV-2 using reverse transcription-polymerase chain reaction (RT-PCR). For negative PCR patients, the presence of other pathogenic respiratory virus was detected by mNGS.

Mick et al. [20] performed pairwise differential expression (DE) analysis between the three patient groups, gene set enrichment analyses (GSEA) on the genes differentially expressed and constructed parsimonious classifiers by combining the LASSO procedure for variable selection and random forest algorithm. They concluded that COVID19 is characterized by markedly attenuated activation of innate immune and pro-inflammatory pathways early in the course of disease compared to other viral ARIs. Human gene counts and metadata are publicity available at https://github.com/czbiohub/covid19-transcriptomics-pathogenesis-diagnostics-results, and IDSeq metagenomic analysis reports are available at https://idseq.net/ under project name “*covid19_transcriptomics_pathogenesis_diagnostics*”.

Before fitting the model, gene counts were variance-stabilizing transformed and patients labels were marked using RT-PCR results for COVID19 patients and mNGS results to distinguish between viral and non-viral ARIs. For more details about the preprocessing step, see Mick et al. [20]. The original set of 15900 features was reduced to the $k=2187$ most correlated genes with the class distinction using Pearson correlation coefficient.

As discussed in Sect. 1, mNGS data are very sensitive to contamination during collection and may lead to contaminated observations of the explanatory variables. Moreover, standard RT-PCR risk of false-positive or false-negative outcomes, and therefore, some observations may be mislabelled. In order to evaluate the performance of the logistic classifier under contamination both in gene expression profiling (leverage points) and mislabelled observations, we introduce both types of data contamination. For the first, we flag a subset of significant variables using standard LASSO and we randomly select a $5\%$ of the observations. For each selected COVID19 observation, we add twice the mean of the variable across all data to significant variables with negative regression coefficient and subtract the same amount to variables with positive regression coefficient. Then, we apply the inverse transformation to the rest of outliers observations. Finally, to generate mislabelled observations, we randomly select a $10\%$ of the sample and switch its label.

## Experiments and Results

We compare the performance of the DPD-based methods with the classical MLE through different accuracy measures, namely sensitivity (true positive rate, TP), specificity (true negative rate, TN) and correct classification rate (CCR). The explicit formulas are

10 $$\begin{aligned} \begin{aligned} \text {TP}&= \frac{\text {true positives}}{(\text {true positives}+ \text {true negatives})}, \\ \text {TN}&= \frac{\text {true negatives}}{(\text {true negatives}+ \text {false positives})} ,\\ \text {CCR}&= \frac{\text {true negatives} + \text {true negatives} }{n}. \end{aligned} \end{aligned}$$

We also report the number of genes selected (model size (MS)) with each of the methods. We fit the preprocessed data to the logistic regression model and use the LASSO and adaptive LASSO (Ad-LASSO) methods to estimate the regression parameters, jointly with our proposed MDPDE for the values $\alpha = 0.1, 0.3, 0.5, 0.7$ and 1, and two different weights functions; $w(s) = 1/s$ yielding to the adaptive DPD-based (Ad-DPD-LASSO) estimator and

$$\begin{aligned} w(s) = {\text {I}}(s\le \lambda ) + \frac{(a\lambda -s)_{+}}{(a-1)\lambda }{\text {I}}(s>\lambda ), \end{aligned}$$

with $a=3.7,$ for the AW-DPD-LASSO estimator. Moreover, we apply the high-dimensional adaptation of the Generalized Information Criterion (HGIC), introduced in Konishi and Kitagawa [19], to select the optimal value of $\lambda$ in (9), given by

11 $$\begin{aligned} \lambda ^*= {\text {min}} \left[ \frac{ -2\log {\mathcal {L}}(\widehat{\varvec{\beta }}_\lambda )}{n} + \frac{\log \log (n)\log (p)}{n}\parallel \widehat{\varvec{\beta }}\parallel _0 \right] \end{aligned}$$

where ${\mathcal {L}}(\widehat{\varvec{\beta }}_\lambda )$ is the logistic loglikelihood function. Since the loss function associated with the logistic regression model is bounded, the penalized estimators are very sensitive to the choice of the penalty parameter. Larger choices of $\lambda$ induce very shrunk estimators. Therefore, $\lambda$ is chosen over a pre-defined bounded grid of values.

To compute the LASSO and Ad-LASSO methods, we use the R package *glmnet*, AW-LASSO is fitted using *ncvreg* package and we fit the DPD-based estimators with our own implemented code available at https://github.com/MariaJaenada/awDPDlasso. Finally, to examine the robustness of the methods, the logistic regression model is fitted with original and contaminated data and then, evaluated with the original data (without outliers) in both settings.

Further, to assess the dependence of the model on the data, we fit the logistic model with the whole dataset and with 5 subsamples containing all observations except for 5 predefined folds, and we report the accuracy measures of the model fitted with the whole data, and the mean of the measures produced by the 5 different models fitted with each fold. The last one allows us to better assess the estimation dependency of the data, since some subsamples would contain more outlier observations than others.

### Diagnosis of COVID-19

We first examine the performance of the different methods when differentiating COVID19 patients from patients suffering other viral and non-viral disease. Table 1 shows the results for the logistic model with two classes, COVID19 patients $(Y=1)$ and the rest of the patients $(Y=0)$ without and with contaminated observations, respectively, with a cut-off of 0.5. It is straightforward to see that adaptive methods select more parsimonious models, but remain competitive to the LASSO and Ad-LASSO in all accuracy measures. The proposed robust methods perform similarly to the LASSO and adaptive LASSO in the absence of contamination, so the penalized DPD-based estimators are competitive to penalized MLEs in a contamination-free scenario. Conversely, DPD-based robust methods maintain high classification rates when contamination is introduced into the data, unlike least squares-based methods, whose sensitivity drops considerably. That is, likelihood methods encounter more difficulties in correctly diagnosing the disease. The effect of contamination is even more pronounced when the fivefold cross-validation dataset is used, as the percentage of outlier observations is higher depending on the fold with lower sample size.

**Table 1:** Accuracy measures when training the logistic regression model with uncontaminated data

	Training with all data				Training with subsamples			
	MS	Rate	TP	TN	MS	Rate	TP	TN
*Fitted with uncontaminated data*								
LASSO	24	0.950	0.926	0.965	18.400	0.908	0.872	0.932
Ad LASSO	9	0.929	0.904	0.944	7.600	0.903	0.868	0.925
AW-LASSO	24	0.929	0.904	0.944	7.600	0.903	0.868	0.925
Ad DPD-LASSO $\alpha$ = 0.1	12	0.954	0.936	0.965	9.8	0.932	0.915	0.943
Ad DPD-LASSO $\alpha$ = 0.3	11	0.954	0.936	0.965	9.6	0.937	0.915	0.951
Ad DPD-LASSO $\alpha$ = 0.5	9	0.950	0.915	0.972	9.0	0.935	0.917	0.947
Ad DPD-LASSO $\alpha$ = 0.7	9	0.950	0.915	0.972	9.2	0.940	0.915	0.957
Ad DPD-LASSO $\alpha$ = 1	9	0.950	0.915	0.972	7.8	0.939	0.911	0.957
AW DPD-LASSO $\alpha$ = 0.1	18	0.958	0.947	0.965	11.8	0.930	0.909	0.944
AW DPD-LASSO $\alpha$ = 0.3	18	0.958	0.936	0.972	11.8	0.933	0.913	0.946
AW DPD-LASSO $\alpha$ = 0.5	19	0.958	0.936	0.972	11.6	0.933	0.911	0.947
AW DPD-LASSO $\alpha$ = 0.7	19	0.950	0.926	0.965	11.6	0.934	0.909	0.950
AW DPD-LASSO $\alpha$ = 1	19	0.950	0.926	0.965	11.8	0.935	0.913	0.950
*Fitted with contaminated data*								
LASSO	12	0.761	0.468	0.951	12.800	0.759	0.457	0.956
Ad LASSO	7	0.777	0.553	0.924	6.600	0.781	0.568	0.919
AW-LASSO	7	0.777	0.553	0.924	6.600	0.781	0.568	0.919
Ad DPD-LASSO $\alpha$ = 0.1	6	0.845	0.766	0.896	8.0	0.820	0.679	0.912
Ad DPD-LASSO $\alpha$ = 0.3	6	0.845	0.766	0.896	7.8	0.785	0.545	0.942
Ad DPD-LASSO $\alpha$ = 0.5	6	0.840	0.755	0.896	7.0	0.816	0.662	0.917
Ad DPD-LASSO $\alpha$ = 0.7	6	0.840	0.755	0.896	7.0	0.787	0.549	0.942
Ad DPD-LASSO $\alpha$ = 1	6	0.845	0.766	0.896	6.6	0.813	0.660	0.914
AW DPD-LASSO $\alpha$ = 0.1	7	0.840	0.745	0.903	6.6	0.821	0.732	0.879
AW DPD-LASSO $\alpha$ = 0.3	7	0.840	0.755	0.896	6.8	0.829	0.747	0.882
AW DPD-LASSO $\alpha$ = 0.5	7	0.845	0.766	0.896	6.4	0.822	0.730	0.882
AW DPD-LASSO $\alpha$ = 0.7	7	0.845	0.766	0.896	6.4	0.817	0.717	0.882
AW DPD-LASSO $\alpha$ = 1	7	0.840	0.745	0.903	6.0	0.814	0.711	0.882

**Fig. 1:**
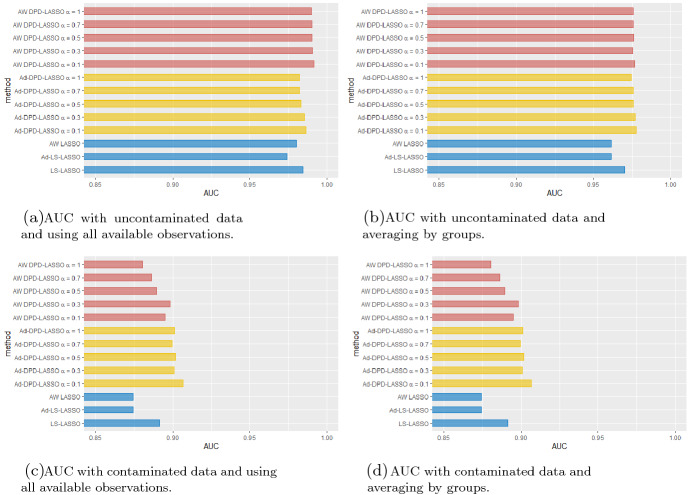
AUC for the different methods with uncontaminated (top) and contaminated (bottom) data

We also study the accuracy by fitting a Receiver Operating Characteristic (ROC) curve of the model and reporting its area under the curve (AUC). The AUC is a robust overall measure to evaluate the performance of score classifiers because its calculation relies on the complete ROC curve and thus involves all possible classification thresholds. Figure 1 shows the AUC for the different methods with uncontaminated (top) and contaminated (bottom) datasets. All methods have a similar performance in the absence of contamination, but when outliers are introduced the AUC of the classical penalized MLEs decreases more than the robust method’s AUC.

**Fig. 2:**
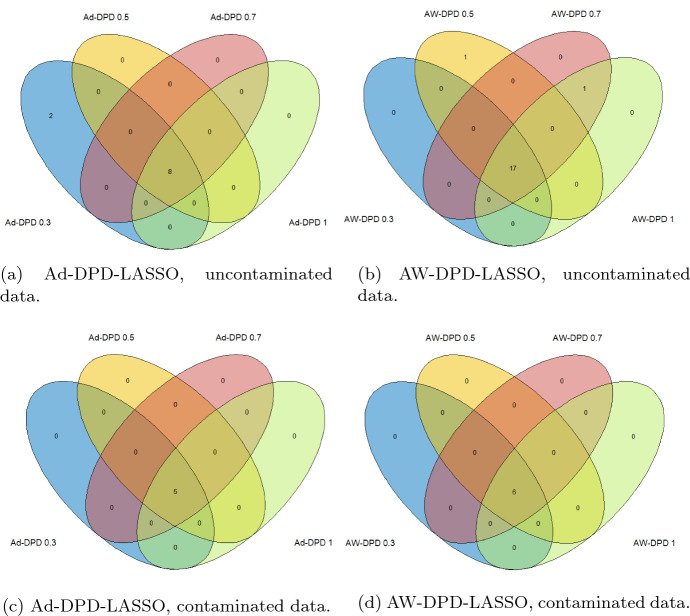
Venn diagrams of gene sets selected by penalized DPD-based methods for different values of $\alpha$ under uncontaminated and contaminated data

**Fig. 3:**
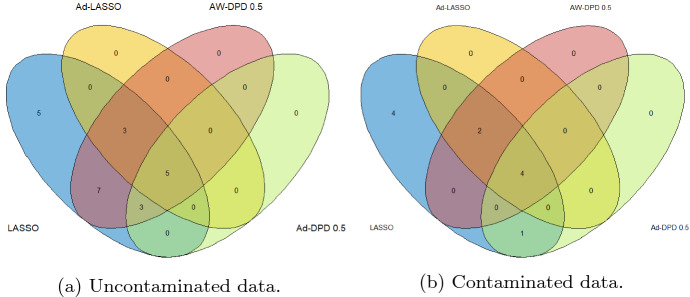
Venn diagrams of gene sets selected by different methods under uncontaminated and contaminated data

Complementary to the accuracy study of the model, it is also interesting to examine common genes selected in each method, and the stability in the selection, in the case of DPD loss-based methods by varying the parameter $\alpha .$ Figure 2 shows Venn diagrams with the number of common genes selected by the DPD-based methods for different values of $\alpha$ under pure (top) and contaminated (bottom) data and the two proposed penalties, adaptive LASSO (right) and adaptive weighted LASSO (right) based on the nonconcave SCAD penalty. As shown, the genes selected with each of the penalties coincide for almost all values of the tuning parameter, showing stability in the variable selection when the value of $\alpha$ is changed. On the other hand, adaptive methods based on the MLE generally shrink the set of selected variables by the standard LASSO procedure. In this case, LASSO and AW-LASSO methods identified the same set of genes under uncontaminated data, whereas Ad-LASSO selects a subset of them, but under contaminated data both Ad-LASSO and AW-LASSO methods pick the same subset of genes selected by the standard LASSO. In contrast, the sets of selected genes vary slightly when fitting the model using different combinations of loss and penalty functions. Figure 3 shows Venn diagrams of the gene sets selected by different methods under pure and contaminated data. It is striking that all methods select over 4–5 common genes, and almost all genes selected by Ad-DPD-LASSO are also selected by the AW-DPD-LASSO method. Following Mick et al. [20], over 10 genes may be enough to construct a competitive classifier for COVID19 diagnosis, and RT-PCR assays usually employ some of the four gene targets, namely ORF1ab/RdRp, E (envelope), N (nucleocapsid), and S (spike) genes for SARS-CoV-2 detection.

**Fig. 4:**
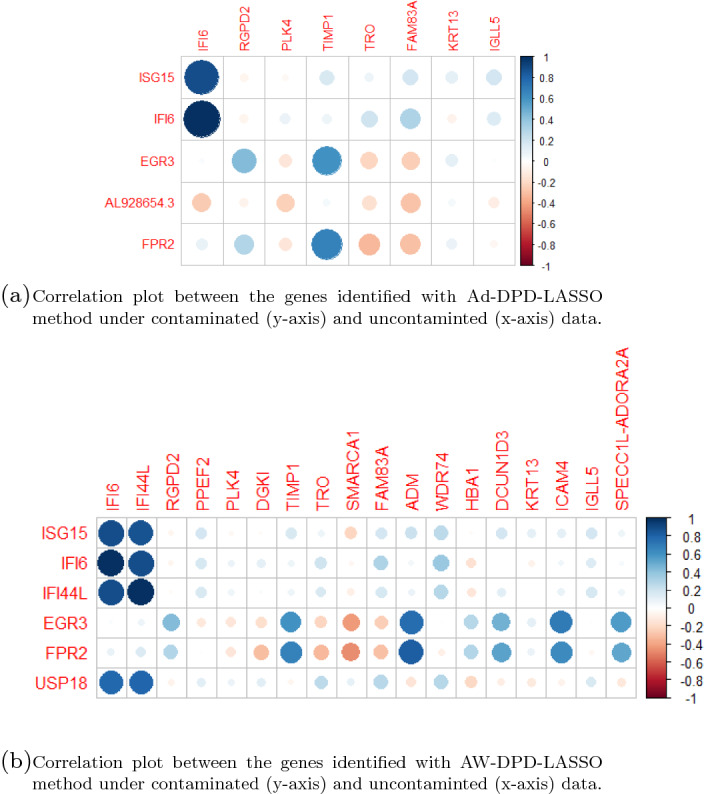
Correlation between the genes identified with DPD-based methods

Conversely, selected genes changed when contaminating the data even when fitting the model with the same estimating method, as our contamination scheme uses the set of important variables to introduce leverage points. Nonetheless, both sets of genes are highly correlated, as shown in Fig. 4. In particular, almost all genes selected under contaminated data are highly correlated with (at least) one gene selected in the absence of contamination.

**Table 2:** Estimated coefficients and OR associated with the selected genes with adaptive penalized DPD-based methods

Gene name	Coef.	OR	Coef.	OR	Coef.	OR	Coef.	OR	Coef.	OR
	$\alpha$ = 0.1		$\alpha$ = 0.3		$\alpha$ = 0.5		$\alpha$ = 0.7		$\alpha$ = 1	
*Ad-DPD-LASSO*										
IFI6	1.42	4.14	1.75	5.76	1.22	3.37	1.27	3.56	1.31	3.71
RGPD2	$-$ 0.52	0.59	$-$ 0.71	0.49	$-$ 0.47	0.63	$-$ 0.49	0.61	$-$ 0.51	0.60
PLK4	0.50	1.64	0.68	1.97	0.48	1.62	0.52	1.68	0.55	1.74
DGKI	0.27	1.32	0.44	1.55	–	–	–	–	–	–
TIMP1	$-$ 1.12	0.33	$-$ 1.37	0.25	$-$ 1.17	0.31	$-$ 1.21	0.30	$-$ 1.25	0.29
TRO	0.74	2.10	0.85	2.35	0.80	2.22	0.81	2.25	0.80	2.23
FAM83A	0.57	1.76	0.92	2.51	0.57	1.76	0.61	1.84	0.67	1.95
KRT13	$-$ 0.33	0.72	$-$ 0.44	0.65	$-$ 0.21	0.81	$-$ 0.23	0.79	$-$ 0.27	0.76
IGLL5	0.33	1.40	0.43	1.53	0.20	1.23	0.22	1.25	0.26	1.30
SPECC1L-ADORA2A	$-$ 0.34	0.71	$-$ 0.42	0.66	–	–	–	–	–	–
HBA1	$-$ 0.25	0.78	–	–	–	–	–	–	–	–
*AW-DPD-LASSO*										
IFI6	1.25	3.49	1.41	4.10	1.48	4.40	0.95	2.59	1.00	2.71
IFI44L	0.31	1.37	0.41	1.50	0.57	1.77	0.26	1.29	0.28	1.32
RGPD2	$-$ 0.45	0.64	$-$ 0.59	0.55	$-$ 0.68	0.51	$-$ 0.43	0.65	$-$ 0.45	0.64
PPEF2	0.52	1.69	0.63	1.89	0.72	2.05	0.42	1.52	0.44	1.56
PLK4	0.79	2.20	0.97	2.64	1.26	3.54	0.58	1.78	0.63	1.87
DGKI	0.55	1.73	0.77	2.17	0.91	2.47	0.46	1.58	0.49	1.63
TIMP1	$-$ 0.97	0.38	$-$ 1.11	0.33	$-$ 1.15	0.32	$-$ 0.86	0.42	$-$ 0.90	0.41
TRO	0.42	1.52	0.49	1.63	0.50	1.64	0.34	1.41	0.36	1.43
FAM83A	0.60	1.82	0.73	2.08	0.70	2.02	0.49	1.63	0.51	1.67
ADM	0.32	1.37	0.43	1.54	0.65	1.92	0.23	1.26	0.27	1.31
WDR74	0.17	1.19	0.18	1.20	0.27	1.31	0.16	1.17	0.17	1.18
HBA1	$-$ 0.40	0.67	$-$ 0.49	0.61	$-$ 0.68	0.51	$-$ 0.18	0.84	$-$ 0.21	0.81
DCUN1D3	$-$ 0.03	0.97	$-$ 0.04	0.96	$-$ 0.19	0.83	$-$ 0.04	0.96	$-$ 0.06	0.94
KRT13	$-$ 0.37	0.69	$-$ 0.48	0.62	$-$ 0.59	0.55	$-$ 0.30	0.74	$-$ 0.33	0.72
ICAM4	$-$ 0.24	0.78	$-$ 0.43	0.65	$-$ 0.55	0.58	$-$ 0.28	0.76	$-$ 0.30	0.74
IGLL5	0.39	1.48	0.47	1.60	0.60	1.81	0.28	1.32	0.31	1.36
SPECC1L-ADORA2A	$-$ 0.43	0.65	$-$ 0.53	0.59	$-$ 0.70	0.50	$-$ 0.26	0.77	$-$ 0.29	0.74
SMARCA1	–	–	–	–	0.26	1.30	–	–	–	–
AL928654.3	–	–		–	–	–	$-$ 0.05	0.95	$-$ 0.04	0.96

One may be also interested in determining the constant effect of a gene on the likelihood that one outcome will occur. Odds ratios (OR) may be used to compare the relative odds of the occurrence of the disease given the expression level of a certain gene. The Odds can be interpreted as the risk or importance of a gene in the diagnostic, so they allow comparison of the magnitude of the risk entailed by different genes for the COVID19 disease. Accordingly, each regression coefficient associated with a gene can be interpreted as the estimated relative increase in the log odds of the outcome per unit increase in the level of that gene. Then, the exponential function of the regression coefficient is the odds ratio associated with a one-unit increase in the expression level. Of course, zero-estimated coefficients, resulting in unit OR, imply that these genes do not affect to the diagnose. Table 2 reports the estimated coefficients and associated OR of the selected genes with the different DPD-based methods. Estimated coefficient and associated OR for the penalized MLE are presented in the Appendix for the seek of briefly. When the model is fitted using penalized MLEs, the OR associated with the selected variables are generally very close to the unit, implying a low importance in the diagnosis. Genes *IFI6* and *IF44L* have the greatest OR value in adaptive methods and standard LASSO, respectively. Those genes are two of the most statistically significant genes upregulated by SARS-CoV-2, according to Mick et al. [20]. In contrast, ORs associated with coefficients obtained using DPD-based methods are generally more distant from the unit, suggesting genes with greater relevance in the diagnosis, including *IFI6* and *IF44L*. In addition, DPD-based methods find some other important genes in the classification. Besides *IFI6* and *IF44L* genes, *TIMP1*, *FAM83A* *TRO* and *WDR74* have been flagged to be specifically upregulated in COVID-19 patients compared to both other viral and non-viral ARIs according to [20, 29]. In turn, *TIMP1* has been shown to be related to SARS-CoV-2 infection with lower expression level during pathogenesis ([24]), which is translated in a low OR of the associated coefficient.

**Table 3:** Accuracy measures when training the logistic regression model with uncontaminated data for the problem of differentiating between covid19 and other virus

	Training with all data				Training with subsamples			
	MS	Rate	TP	TN	MS	Rate	TP	TN
*Fitted with uncontaminated data*								
LASSO	17	0.919	0.989	0.756	12.600	0.902	0.977	0.732
Ad LASSO	6	0.904	0.968	0.756	6.800	0.911	0.957	0.805
AW-LASSO	23	0.956	0.989	0.878	6.800	0.911	0.957	0.805
Ad DPD-LASSO $\alpha$ = 0.1	8	0.963	0.968	0.951	8.600	0.932	0.957	0.873
Ad DPD-LASSO $\alpha$ = 0.3	10	0.970	0.989	0.927	7.800	0.930	0.968	0.844
Ad DPD-LASSO $\alpha$ = 0.5	10	0.970	0.989	0.927	7.600	0.935	0.974	0.844
Ad DPD-LASSO $\alpha$ = 0.7	10	0.970	0.989	0.927	7.400	0.933	0.972	0.844
Ad DPD-LASSO $\alpha$ = 1	8	0.956	0.979	0.902	6.800	0.939	0.968	0.873
AW DPD-LASSO $\alpha$ = 0.1	9	0.963	0.979	0.927	9.600	0.947	0.972	0.888
AW DPD-LASSO $\alpha$ = 0.3	10	0.963	0.979	0.927	9.800	0.945	0.968	0.893
AW DPD-LASSO $\alpha$ = 0.5	10	0.963	0.979	0.927	9.000	0.942	0.970	0.878
AW DPD-LASSO $\alpha$ = 0.7	10	0.963	0.979	0.927	8.800	0.942	0.970	0.878
AW DPD-LASSO $\alpha$ = 1	10	0.963	0.979	0.927	9.200	0.932	0.964	0.859
*Fitted with contaminated data*								
LASSO	5	0.807	0.989	0.390	3.2	0.750	0.991	0.195
Ad LASSO	4	0.844	0.957	0.585	2.2	0.753	0.989	0.210
AW-LASSO	4	0.844	0.957	0.585	2.2	0.753	0.989	0.210
Ad DPD-LASSO $\alpha$ = 0.1	5	0.881	0.926	0.780	3.4	0.760	0.974	0.268
Ad DPD-LASSO $\alpha$ = 0.3	5	0.881	0.926	0.780	3.0	0.759	0.974	0.263
Ad DPD-LASSO $\alpha$ = 0.5	5	0.807	0.989	0.390	3.0	0.759	0.974	0.263
Ad DPD-LASSO $\alpha$ = 0.7	5	0.807	0.989	0.390	3.0	0.759	0.974	0.263
Ad DPD-LASSO $\alpha$ = 1	5	0.807	0.989	0.390	3.0	0.759	0.974	0.263
AW DPD-LASSO $\alpha$ = 0.1	5	0.904	0.947	0.805	9.2	0.855	0.879	0.800
AW DPD-LASSO $\alpha$ = 0.3	5	0.904	0.947	0.805	7.6	0.855	0.883	0.790
AW DPD-LASSO $\alpha$ = 0.5	5	0.904	0.947	0.805	6.4	0.870	0.906	0.785
AW DPD-LASSO $\alpha$ = 0.7	5	0.904	0.947	0.805	4.8	0.862	0.923	0.722
AW DPD-LASSO $\alpha$ = 1	5	0.904	0.947	0.805	4.4	0.865	0.930	0.717

**Table 4:** Estimated coefficients and OR associated with the selected genes with adaptive penalized DPD-based methods for differentiating between viral ARIs

Gene name	Coef.	OR	Coef.	OR	Coef.	OR	Coef.	OR	Coef.	OR
	$\alpha$ = 0.1		$\alpha$ = 0.3		$\alpha$ = 0.5		$\alpha$ = 0.7		$\alpha$ = 1	
*Ad-DPD-LASSO*										
LGR6	1.17	3.23	0.74	2.10	0.76	2.15	0.88	2.41	0.89	2.43
TIMP1	$-$ 1.26	*0.28*	$-$ 0.85	0.43	$-$ 0.87	0.42	$-$ 1.01	0.36	$-$ 0.93	0.40
TRO	2.17	*8.75*	1.61	4.98	1.58	4.85	1.60	4.95	1.74	5.69
SMARCA1	1.13	*3.10*	1.13	3.09	1.09	2.98	1.04	2.83	1.21	3.35
WDR74	0.79	*2.20*	0.90	2.45	1.04	2.82	0.91	2.48	1.10	2.99
AL928654.3			$-$ 0.42	0.66	$-$ 0.49	0.61	$-$ 0.27	0.76	$-$ 0.47	0.62
ICAM4	$-$ 0.72	*0.49*	$-$ 0.74	0.48	$-$ 0.87	0.42	$-$ 0.88	0.41	$-$ 0.96	0.38
IGLL5	0.03	1.03	0.11	1.12	0.05	1.06	–	–	0.06	1.06
GSTA2			0.34	1.40	0.34	1.40	–	–	0.30	1.35
*AW-DPD-LASSO*										
LGR6	1.26	3.52	0.44	1.55	0.48	1.61	0.53	1.70	0.67	1.96
GSTA2	0.35	1.42	0.54	1.71	0.59	1.80	0.63	1.88	0.77	2.17
TRO	1.90	6.68	0.82	2.28	0.92	2.51	1.03	2.81	1.44	4.23
SMARCA1	1.36	3.90	1.23	3.41	1.34	3.82	1.46	4.31	1.85	6.37
WDR74	0.99	2.69	0.62	1.86	0.67	1.95	0.72	2.05	0.85	2.33
IGLL5	0.38	1.47	0.40	1.48	0.43	1.54	0.47	1.59	0.56	1.76
TIMP1	$-$ 1.26	0.28	–	–	–	–	–	–	–	–
ICAM4	$-$ 0.90	0.41	–	–	–	–	–	–	–	–
PLEK	–	–	$-$ 0.16	0.85	$-$ 0.18	0.84	$-$ 0.20	0.82	$-$ 0.23	0.79
PDGFRB	–	–	$-$ 0.27	0.77	$-$ 0.28	0.76	$-$ 0.28	0.75	$-$ 0.29	0.75
PCSK5	–	–	0.03	1.03	0.03	1.03	0.04	1.04	0.04	1.04

The identified genes in our analysis mostly coincide with some biomarkers discussed in the literature. In particular, 20 of the 23 genes identified using LASSO penalized MLE were also selected in Mick et al. [20] classifier, which uses a total of 27 genes. However, we have further explored the importance given by the classifier to each gene, studied the stability of the model when varying the penalty and, in the case of DPD-based estimators, the tuning parameter $\alpha .$ Adaptive methods fit more parsimonious models, while DPD-based method gives more importance of the selected genes. These two properties can be of great use when diagnosis new patients. Conversely, the results should be understood with caution, as the sample size of the data is not large enough and the conclusions may not be generalizable. Nonetheless, we can draw from the study the usefulness of penalized DPD-based methods, which perform well in the absence of contamination and increase the accuracy of the model when the data are contaminated, which is quite common when dealing with mNGS data.

### Differentiating Between Viral ARIs

The previous results were calculated with the aggregated data, where the class NO-COVID19 included viral and non-viral ARIs. We now study the performance of the logistic model in differentiating COVID19 from other viral diseases. In this case, only $n=135$ observations are available. We contaminate the data using the same methodology as described in Sect. 4, and we fit the model using all available observations and a fivefolds separately. Table 3 shows the accuracy measures produced by different methods under uncontaminated and contaminated scenario, respectively. Again, the proposed DPD-based estimators perform competitively in the absence of contamination and clearly improves the stability of the estimators when using the weighted adaptive LASSO. The decrease in specificity in the contaminated scenario stands out when dividing the sample in fivefolds. In this case, the sample size decreases to $n=108$ observations, which increases sensitivity to outliers. Classical likelihood-based methods diagnose mostly all patients having COVID19 and is unable to differentiate it from other ARIs. On the contrary, our DPD-based robust adaptive weighted methods maintain a sufficiently high specificity and sensitivity when training with the whole dataset, proving their ability to differentiate between different viruses. However, when a too reduced training set is used, the adaptive DPD-LASSO method is highly dependent on the initial value and performs worse than the adaptively weighted method, which in turn maintain competitive classification rates. This classification problem illustrates the advantage of the robust procedure for high dimensional classification.

Table 4 presents the estimated coefficients and associated OR of the selected genes with DPD-based methods. The results for penalized MLEs are reported in the Appendix. Again, robust methods give more importance to the selected genes, which is shown by their associated OR, and most of the selected genes with the adaptive and weighted adaptive penalties match. Genes selected by DPD-based methods for differentiating between ARIs were mostly identified when distinguishing COVID 19 from other diseases. In particular, *TIMP1*, *TRO*, *WDR74*, *AL928654.3,* *ICAM4*, and *IGLL5* were also identified by at least one of the DPD-based methods, and *LGR6,* *SMARCA1* and *PCSK5* were identified by the classical LASSO estimator.

## Conclusions

Robust penalized logistic regression is specially convenient when dealing with gene-based classification problems. From our results, DPD-based robust methods outperform classical ones when training data are contaminated with leverage points or mislabelled observations, which is quite common in genetic datasets. In particular, when the MLE is used, correct diagnosis of the COVID19 is highly affected by this data contamination and only a $50\%$ of sensitivity is achieved. Thus, the model is useless for the diagnosis of new patients. Conversely, the robust DPD-based methods achieve highest sensitivity rates in contaminated scenarios and are competitive in the absence of contamination, presenting a compelling proposal. Besides, gene selection stability is shown within the same DPD-based penalized estimators family, and identified genes with robust methods play a more important role in the diagnosis than genes selected by non-robust estimators.

The accuracy loss of the non-robust methods under data contamination is emphasized when differentiating between viral ARIs. In this scenario, penalized MLEs lose their ability to detect viral diseases other than COVID, whereas robust estimators manage to maintain sufficiently high specificity. Weighted adaptive DPD-based estimators show the best performance in this case. All these results presented in this paper illustrate the benefit of using the robust DPD-based procedures which can be used routinely in any future real-life analysis of high-dimensional gene expression data and associated classification problems.

## A Additional Results

Tables 5 and 6 present the estimated coefficients and associated OR of the penalized MLEs with LASSO, Ad-LASSO and AW-LASSO penalties, for the diagnosis of COVID19 patients and differentiating between ARIs classification problems, respectively.

**Table 5:** Estimated coefficients and OR associated with the selected genes with penalized MLE methods

Gene name	LASSO		Ad-LASSO		AW-LASSO	
	Coef.	OR	Coef.	OR	Coef.	OR
IFI6	$-$ 0.02	0.98	1.02	2.78	0.71	2.03
LGR6	0.28	1.33	0.04	1.04	0.00	1.00
RGPD2	$-$ 0.27	0.76	$-$ 0.01	0.99	$-$ 0.18	0.83
PPEF2	0.04	1.04	0.08	1.09	0.07	1.07
PLK4	$-$ 0.01	0.99	0.02	1.02	0.08	1.08
TIMP1	$-$ 0.03	0.97	$-$ 0.49	0.62	$-$ 0.36	0.70
TRO	0.02	1.02	0.65	1.92	0.22	1.24
SMARCA1	0.00	1.00	0.23	1.26	0.04	1.04
FAM83A	$-$ 0.00	1.00	0.08	1.08	0.09	1.10
DCUN1D3	$-$ 0.12	0.88	$-$ 0.09	0.92	$-$ 0.17	0.85
ICAM4	0.02	1.02	$-$ 0.03	0.97	$-$ 0.16	0.85
GPR153	–	–	$-$ 0.02	0.98	–	–
H2AC20	–	–	0.28	1.33	–	–
GLUL	–	–	$-$ 0.27	0.76	–	–
AFF1	–	–	$-$ 0.03	0.97	–	–
CASP3	–	–	0.00	1.00	–	–
RNF39	-	–	$-$ 0.00	1.00	–	–
CDKN1A	–	–	$-$ 0.23	0.80	–	–
FBXW2	–	–	$-$ 0.12	0.88	–	–
RALGDS	–	–	$-$ 0.01	0.99	–	–
TOLLIP	–	–	$-$ 0.08	0.92	–	–
BORCS7	–	–	0.06	1.06	–	–
CKAP2	–	–	0.02	1.02	–	–
IFI44L	1.02	2.78	–	–	0.09	1.09
DGKI	0.08	1.09	–	–	0.07	1.07
PCSK5	$-$ 0.23	0.80	–	–	0.01	1.01
ADM	$-$ 0.49	0.62	–	–	$-$ 0.08	0.92
WDR74	0.65	1.92	–	–	0.22	1.24
AL928654.3	0.23	1.26	–	–	$-$ 0.09	0.92
HBA1	0.08	1.08	–	–	$-$ 0.05	0.95
EIF3CL	$-$ 0.01	0.99	–	–	$-$ 0.02	0.98
KRT13	$-$ 0.08	0.92	–	–	$-$ 0.11	0.90
TGM3	0.06	1.06	–	–	$-$ 0.00	1.00
IGLL5	$-$ 0.09	0.92	–	–	0.11	1.11
SPECC1L-ADORA2A	$-$ 0.03	0.97	–	-	$-$ 0.07	0.93

**Table 6:** Estimated coefficients and OR associated with the selected genes with penalized MLE methods for differentiating between ARIs

Gene name	LASSO		Ad-LASSO		AW-LASSO	
	Coef.	OR	Coef.	OR	Coef.	OR
LGR6	0.20	1.22	0.42	1.52	0.59	1.81
TIMP1	$-$ 0.03	0.98			$-$ 0.28	0.76
TRO	0.20	1.22	0.25	1.29	0.38	1.47
SMARCA1	0.09	1.10			0.14	1.15
WDR74	0.20	1.22	0.26	1.29	0.17	1.19
GPR153	–	–	–	–	$-$ 0.08	0.92
GIPC2	–	–	–	–	0.02	1.02
GLUL	–	–	–	–	$-$ 0.33	0.72
ORC4	–	–	–	–	0.38	1.46
ZSCAN23	–	–	–	–	0.07	1.07
LYRM2	–	–	–	–	0.12	1.13
PMPCB	–	–	–	–	0.09	1.09
CPNE3	–	–	–	–	0.04	1.04
RALGDS	–	–	–	–	$-$ 0.14	0.87
BORCS7	–	–	–	–	0.14	1.15
KIAA0586	–	–	–	–	0.04	1.04
SNRPN	–	–	–	–	0.07	1.08
NUP88	–	–	–	–	0.07	1.07
HS3ST3B1	–	–	–	–	$-$ 0.09	0.92
R3HDM4	–	–	–	–	$-$ 0.06	0.94
TRIP10	–	–	–	–	$-$ 0.03	0.97
TPM4	–	–	–	–	$-$ 0.03	0.97
PLEK	$-$ 0.20	0.82	$-$ 0.24	0.79	–	–
GSTA2	0.27	1.31	0.36	1.43	–	–
PDGFRB	$-$ 0.07	0.93	–	–	–	-
NRSN1	0.11	1.11	–	–		–
PCSK5	0.06	1.06	–	–	–	–
HCAR2	$-$ 0.03	0.97	–	–	–	–
AL928654.3	$-$ 0.02	0.98	–	–	–	–
DCUN1D3	$-$ 0.04	0.96	–	–		–
KRT13	$-$ 0.01	0.99	–	–	–	–
ICAM4	$-$ 0.03	0.97	–	–	–	–
IGLL5	0.08	1.08	–	–	–	–
